# Innate Lymphoid Cells and T Cells Contribute to the Interleukin‐17A Signature Detected in the Synovial Fluid of Patients With Juvenile Idiopathic Arthritis

**DOI:** 10.1002/art.40731

**Published:** 2019-01-28

**Authors:** Elizabeth C. Rosser, Hannah Lom, David Bending, Chantal L. Duurland, Mona Bajaj‐Elliott, Lucy R. Wedderburn

**Affiliations:** ^1^ University College London Great Ormond Street Institute of Child Health and Arthritis Research UK Centre for Adolescent Rheumatology at University College London University College London Hospitals, and Great Ormond Street Hospital London UK; ^2^ Institute of Immunology and Immunotherapy College of Medical and Dental Sciences University of Birmingham Birmingham UK; ^3^ University College London Great Ormond Street Institute of Child Health London UK; ^4^ University College London Great Ormond Street Institute of Child Health, Arthritis Research UK Centre for Adolescent Rheumatology at University College London, University College London Hospitals, and Great Ormond Street Hospital, and NIHR Biomedical Research Centre at Great Ormond Street Hospital London UK; ^5^ Present address: Leiden University Medical Center, Leiden, The Netherlands

## Abstract

**Objective:**

Evidence suggests that aberrant function of innate lymphoid cells (ILCs), whose functional and transcriptional profiles overlap with those of Th cell subsets, contributes to immune‐mediated pathologies. To date, analysis of juvenile idiopathic arthritis (JIA) immune pathology has concentrated on the contribution of CD4+ T cells; we have previously identified an expansion of Th17 cells within the synovial fluid (SF) of JIA patients. We undertook this study to extend this analysis to further investigate the role of ILCs and other interleukin‐17 (IL‐17)–producing T cell subsets in JIA.

**Methods:**

ILCs and CD3+ T cell subsets were defined in peripheral blood mononuclear cells (PBMCs) from healthy adults, healthy children, and JIA patients and in SF mononuclear cells (SFMCs) from JIA patients using flow cytometry. Defined subsets in SFMCs were correlated with clinical measures including physician's global assessment of disease activity on a visual analog scale, number of joints with active disease, and erythrocyte sedimentation rate. Transcription factor and cytokine profiles of sorted ILCs were assessed by quantitative reverse transcriptase–polymerase chain reaction.

**Results:**

Group 1 ILCs (ILC1s), NKp44− group 3 ILCs (natural cytotoxicity receptor–negative [NCR−] ILC3s), and NKp44+ ILC3s (NCR+ ILC3s) were enriched in JIA SFMCs compared to PBMCs, which corresponded to an increase in transcripts for *TBX21*,* IFNG*, and *IL17A*. Of the ILC subsets, the frequency of NCR− ILC3s in JIA SFMCs displayed the strongest positive association with clinical measures, which was mirrored by an expansion in IL‐17A+CD4+, IL‐17A+CD8+, and IL‐17A+ γδ T cells.

**Conclusion:**

We demonstrate that the strength of the IL‐17A signature in JIA SFMCs is determined by multiple lymphoid cell types, including NCR− ILC3s and IL‐17A+CD4+, IL‐17A+CD8+, and IL‐17A+ γδ T cells. These observations may have important implications for the development of stratified therapeutics.

## Introduction

Juvenile idiopathic arthritis (JIA), the most common rheumatic disease in childhood, is characterized by joint inflammation lasting longer than 6 weeks 1. The umbrella term JIA encompasses many subtypes including oligoarticular JIA, polyarticular JIA, enthesitis‐related arthritis (ERA), psoriatic arthritis, and systemic JIA 1. Apart from systemic JIA, which has a distinct pathogenesis, studies suggest that synovitis in a proportion of JIA cases is linked to the interleukin‐23 (IL‐23)/IL‐17A cytokine axis 2. To date, the IL‐17A signature within JIA synovial fluid mononuclear cells (SFMCs) has been delineated only in CD4+ Th cells.

Emerging evidence indicates that innate lymphoid cells (ILCs), the most recently discovered members of the lymphoid family, have critical roles in immunity, tissue development, and remodeling 3. Similar to Th cells, CD127+ helper ILCs can be divided into distinct groups based on their functional and transcriptional profiles. Th1 cell–equivalent group 1 ILCs (ILC1s) express the transcript for *TBX21* (T‐bet) and produce interferon‐γ (IFNγ), Th2 cell–equivalent group 2 ILCs (ILC2s) express the transcript for *GATA3* and produce IL‐13, Th17 cell–equivalent natural cytotoxicity receptor–negative (NCR−) group 3 ILCs (ILC3s) express the transcript for *RORC2* and produce IL‐17A/IL‐22, and Th22 cell–equivalent NCR+ ILC3s express the transcripts for *RORC2* and *AHR* and only produce IL‐22 3. From a clinical viewpoint, chronic ILC activation has been associated with a wide range of inflammatory disorders 3. To date, there are no data regarding ILC phenotype/function in childhood arthritides.

In the present study, we demonstrate that ILC1, NCR− ILC3, and NCR+ ILC3 subsets are expanded within SFMCs of JIA patients compared to peripheral blood mononuclear cells (PBMCs) of healthy control adults, PBMCs of healthy control children, and PBMCs of JIA patients, but that NCR− ILC3s show the strongest association with multiple measures of clinical severity. Notably, the increase in NCR− ILC3s within JIA SFMCs was accompanied by an increase in IL‐17–producing CD4+, CD8+, and γδ T cells. These data suggest that the IL‐17 signature previously observed in CD4+ T cells within JIA SFMCs may extend to the ILC compartment and other T cell subsets.

## Patients and methods

#### Samples from humans

PB from healthy control adults, healthy control children, and JIA patients as well as SF from JIA patients were obtained with fully informed and age‐appropriate consent as approved by the London‐Bloomsbury Research Ethics Committee (ref. no. 95RU04) in accordance with the Declaration of Helsinki. Clinical subtypes of JIA were defined according to International League of Associations for Rheumatology criteria 1. Clinical and demographic data are shown in Supplementary Table 1, available on the *Arthritis & Rheumatology* web site at http://onlinelibrary.wiley.com/doi/10.1002/art.40731/abstract. PBMCs and SFMCs were prepared by density‐gradient centrifugation. Before processing, SF samples were treated with hyaluronidase (10 units/ml; Sigma‐Aldrich) for 30 minutes at 37°C.

#### Flow cytometry, ImageStream analysis, and cell sorting

Flow cytometry was performed using directly conjugated monoclonal antibodies (listed in Supplementary Table 2, http://onlinelibrary.wiley.com/doi/10.1002/art.40731/abstract) as described 2. Dead cells were excluded using Live/Dead discrimination dye (Thermo Scientific). ILCs were defined as cells within the lymphocyte gate that were single cells, lineage negative (defined as CD1a, CD3, CD11c, CD14, CD16, CD19, CD34, CD94, CD123, blood dendritic cell antigen 2, Fcε receptor Iα, αβ T cell receptor [αβ TCR], and γδ TCR–negative), CD45+, CD127+, and CD161+. ILC subpopulations were defined according to phenotype: ILC1s (chemoattractant receptor–like molecule expressed on Th2 cells–negative [CRTH2−] c‐Kit−), ILC2s (CRTH2+), NCR− ILC3s (CRTH2−c‐Kit+NKp44−), and NCR+ ILC3s (CRTH2−c‐Kit+NKp44+) 4. Data were acquired on an LSRII flow cytometer (BD Biosciences) and analyzed using FlowJo software version 10.1 (Tree Star). For ImageStream analysis, ILCs were stained and analyzed on an Amnis ImageStreamX Mark II (Merck Millipore). For cell sorting, ILCs were stained as above and sorted on a FACSAria III (BD Biosciences).

#### RNA extraction and quantitative reverse transcriptase–polymerase chain reaction (qRT‐PCR)

RNA was routinely extracted from sorted ILCs (~50,000**–**100,000) using an Arcturus Picopure RNA isolation kit (ThermoFisher Scientific), and complementary DNA was synthesized using an iScript DNA kit according to the instructions of the manufacturer (Bio‐Rad). Complementary DNA was amplified using SYBR Green Master Mix (Bio‐Rad) with custom primers for *AHR*,* RORC2*,* GATA3*,* TBX21*,* IL17A*,* IL22*,* IL13*, and *IFNG* (Life Technologies); primer sequences are listed in Supplementary Table 3, http://onlinelibrary.wiley.com/doi/10.1002/art.40731/abstract. For each sample, transcript quantity was normalized to β‐actin (*ACTB*) expression.

#### Statistical analysis

Statistical analysis was performed using GraphPad Prism software version 5.03. Data are shown as the mean ± SEM. For comparison of 2 groups, Mann‐Whitney U tests were used. For multiple comparison testing, Kruskal‐Wallis tests with Dunn's multiple comparison tests were performed. *P* values less than 0.05 were considered significant. Spearman correlation with Bonferroni correction for multiple testing was used for correlation analyses, and uncorrected *P* values and the adjusted Bonferroni alpha cutoff are reported in Supplementary Table 4, http://onlinelibrary.wiley.com/doi/10.1002/art.40731/abstract.

## Results

#### Expansion of ILC1s, NCR− ILC3s, and NCR+ ILC3s in JIA SFMCs

**Figure 1:**
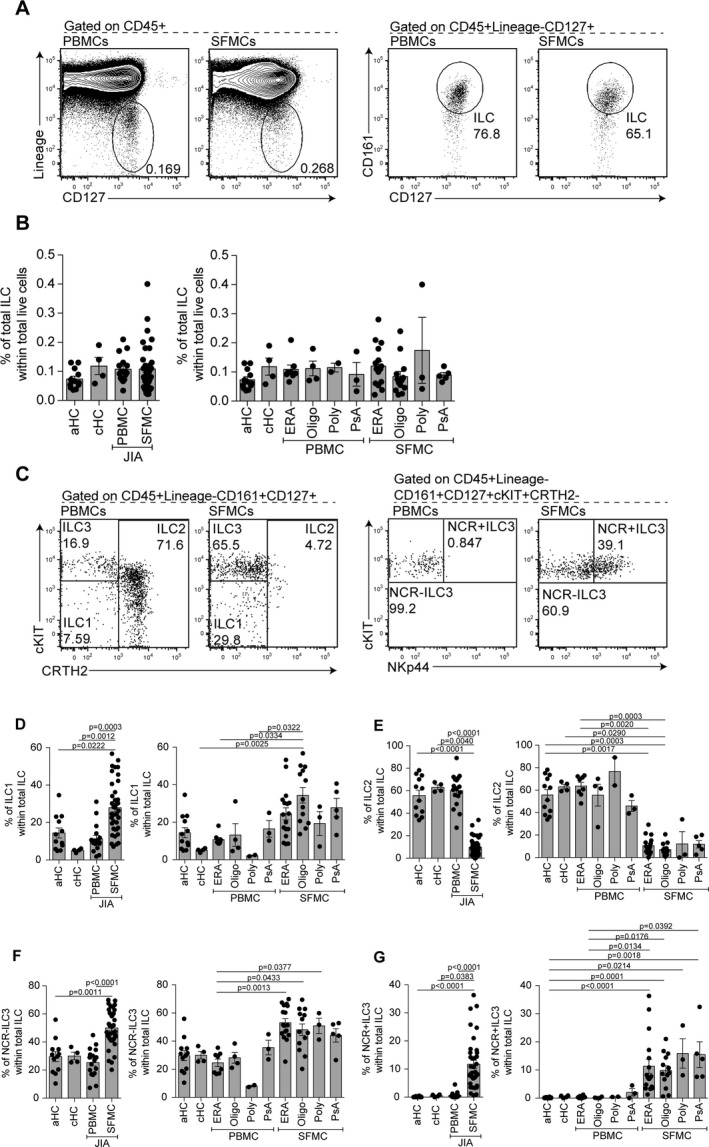
Group 1 innate lymphoid cells (ILC1s), natural cytotoxicity receptor–negative (NCR−) group 3 ILCs (ILC3s), and NCR+ ILC3s are expanded in synovial fluid (SF) of patients with juvenile idiopathic arthritis (JIA). **A** and **B**, Representative flow cytometry plots (**A**) and summary scatterplots with bar charts (**B**) showing the frequency of total ILCs (defined as lineage−CD127+CD161+) within total CD45+ live cells in peripheral blood mononuclear cells (PBMCs) from healthy adults (aHC; n = 12), PBMCs from healthy children (cHC; n = 4), PBMCs from JIA patients (total n = 17; 8 with enthesitis‐related arthritis [ERA], 4 with oligoarticular JIA [Oligo], 2 with polyarticular JIA [Poly], and 3 with psoriatic arthritis [PsA]), and SF mononuclear cells (SFMCs) from JIA patients (total n = 38; 17 with ERA, 13 with oligoarticular JIA, 3 with polyarticular JIA, and 5 with PsA). **C**–**G**, Representative flow cytometry plots (**C**) and summary scatterplots with bar charts (**D**–**G**) showing the frequency of ILC1s (defined as lineage–CD127+CD161+c‐Kit−chemoattractant receptor–like molecule expressed on Th2 cells–negative [CRTH2−]) (**D**), group 2 ILCs (ILC2s; defined as lineage−CD127+CD161+CRTH2+) (**E**), NCR− ILC3s (defined as lineage−CD127+CD161+c‐Kit+CRTH2−NKp44−) (**F**), and NCR+ ILC3s (defined as lineage−CD127+CD161+c‐Kit+CRTH2−NKp44+) (**G**) within total ILCs in PBMCs from healthy adults (n = 12), PBMCs from healthy children (n = 4), PBMCs from JIA patients (total n = 17; 8 with ERA, 4 with oligoarticular JIA, 2 with polyarticular JIA, and 3 with PsA), and SFMCs from JIA patients (total n = 38; 17 with ERA, 13 with oligoarticular JIA, 3 with polyarticular JIA, and 5 with PsA). Symbols represent individual patients; bars show the mean ± SEM. Significance was determined by Kruskal‐Wallis test with Dunn's multiple comparison test.

ILCs (lineage−CD45+CD127+CD161+) were enumerated within PBMCs and SFMCs of JIA patients and within PBMCs of healthy control adults and children (Figures 1A and B). The ILC population was small as a proportion of all live mononuclear cells in both compartments (0.005–0.5% of total live mononuclear cells), consistent with other reports of human adult PBMCs 4. There were no significant differences in the proportions of ILCs within all mononuclear cell populations tested (Figures 1A and B).

Next, we determined whether there were differences in ILC subsets, as a proportion of total ILCs, in PBMCs versus SFMCs. ILC subsets were identified as ILC1s (CRTH2−c‐Kit−), ILC2s (CRTH2+), and ILC3s (CRTH2−c‐Kit+), the last of which were divided into NCR+ and NCR− subgroups according to expression of NKp44. Given the low frequency of ILC populations, ImageStream analysis was used to confirm that ILC subtypes within both PBMCs and SFMCs were lymphoid in morphology and that antibody staining was localized to the membrane prior to analysis (see Supplementary Figure 1A, http://onlinelibrary.wiley.com/doi/10.1002/art.40731/abstract). Assessment of ILC subset frequency showed that ILC1s were significantly enriched as a proportion of total ILCs in JIA SFMCs compared to JIA PBMCs, healthy control adult PBMCs, and healthy control children PBMCs (Figures 1C and D). Further analyses revealed that the largest differences in ILC1 frequency were observed between PBMCs from healthy control children and SFMCs from patients with oligoarticular JIA (Figure 1D), which complements historical data demonstrating that T cells from patients with oligoarticular JIA produce a significant amount of IFNγ 5. ILC2s, which have been implicated in the resolution of chronic joint inflammation by supporting Treg cell function 6, were significantly lower in SFMCs than in PBMCs from patients or controls, with both oligoarticular JIA and ERA displaying a significant difference between SFMCs and healthy control adult PBMCs (Figures 1C and E). These differences may reflect the larger numbers of patients with ERA and oligoarticular JIA analyzed in our cohort.

Within the ILC3 compartment, the proportion of NCR− ILC3s was significantly higher in SFMCs than in healthy control adult PBMCs or JIA PBMCs, and this effect was observed in all JIA subtypes (Figures 1C and F). There was a significant enrichment of NCR+ ILC3s (which were present at very low frequency in PBMCs from patients and controls) in SFMCs from patients with all JIA subtypes compared to healthy control adult PBMCs (Figures 1C and G). No differences were observed in the frequency of ILC subsets among PBMCs or SFMCs between all JIA subtypes analyzed.

#### Altered transcriptional profile of ILCs within JIA SFMCs compared to that of ILCs within PBMCs

**Figure 2:**
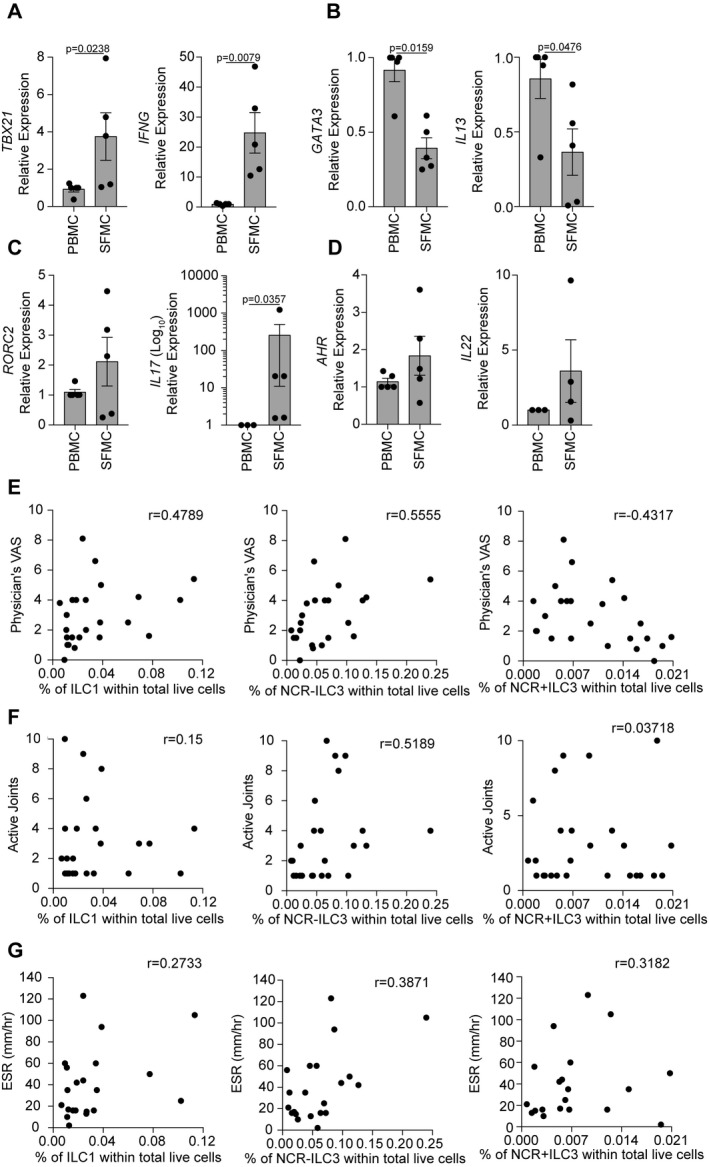
Changes in ILC subset frequency within total ILCs within SFMCs are associated with changes in transcriptional profile and in clinical severity. **A**–**D**, Summary scatterplots with bar charts showing the relative expression of *TBX21* and *IFNG* (**A**), *GATA3* and *IL13* (**B**), *RORC2* and *IL17A* (**C**), and *AHR* and *IL22* (**D**) in SFMCs (n = 5 patients) and PBMCs (n = 5 patients) as measured by quantitative reverse transcriptase–polymerase chain reaction. Symbols represent individual patients; bars show the mean ± SEM. Significance was determined by Mann‐Whitney U tests. **E**–**G**, Scatterplots showing the relationships of the physician's global assessment of disease activity on a visual analog scale (VAS) (n = 24 patients) (**E**), the number of joints with active disease (n = 27 patients) (**F**), and the erythrocyte sedimentation rate (ESR) (n = 18 patients) (**G**) with the frequency of ILC1s, NCR− ILC3s, and NCR+ ILC3s within total live cells within JIA SFMCs at the time of sampling. Significance was determined by Spearman correlation with Bonferroni correction for multiple testing. See Figure 1 for other definitions.

Previously reported studies have demonstrated that ILC1s, ILC2s, NCR− ILC3s, and NCR+ ILC3s express specific transcription factors and cytokine profiles that parallel Th1, Th2, Th17, and Th22 cells, respectively 3, 4. Accordingly, we next assessed whether variation in the frequency of ILC subsets observed in PBMCs versus SFMCs was associated with an altered transcriptional profile. Gene expression analysis of transcription factors (*TBX21*,* GATA3*,* RORC2*,* AHR*) and cytokines (*IFNG*,* IL13*,* IL17A*,* IL22*) by qRT‐PCR in ILCs within SFMCs relative to ILCs within healthy control adult PBMCs demonstrated that there was a significant increase in Th1 cell–associated *TBX21* and *IFNG* and a significant decrease in Th2 cell–associated *GATA3* and *IL13*, mirroring the changes in subset frequency we had observed by flow cytometry (Figures 2A and B). Analysis of transcription factors and cytokines associated with ILC3 subsets demonstrated a significant increase in the relative expression of *IL17* and a trend toward an increase in *RORC2*,* AHR*, and *IL22*, although the expression varied between JIA SFMC samples (Figures 2C and D). Due to the small amount of blood obtained from children, we were unable to isolate sufficient ILCs from healthy control children PBMCs or JIA PBMCs to perform qRT‐PCR analysis.

#### Changes in ILC subset frequency within SF of patients with JIA are associated with disease severity

As the ILC subset frequency and transcriptional profile were altered in JIA SFMCs, we next investigated whether these changes were associated with clinical measures of disease severity. Physician's global assessment of disease activity on a visual analog scale (VAS), number of joints with active disease, and erythrocyte sedimentation rate (ESR) were correlated with the proportions of ILC1s, NCR+ ILC3s, or NCR− ILC3s within total ILCs within SFMCs. ILC2s were not included in these analyses due to their paucity within SFMCs. This exploratory analysis showed promising trends that the frequencies of ILC1, NCR+ ILC3, and NCR− ILC3 subsets were associated with disease severity (Figure 2E; also see Supplementary Table 4, http://onlinelibrary.wiley.com/doi/10.1002/art.40731/abstract). Notably, while ILC1s and NCR− ILC3s were positively associated with an increase on the physician's VAS score, there was a negative correlation between the physician's VAS score and NCR+ ILC3s. One potential hypothesis is that NCR+ ILC3s may also be important in the resolution of joint inflammation in JIA, similar to previously reported observations in the inflamed gastrointestinal tract in ankylosing spondylitis 7.

We next assessed potential correlations between ILC subset frequency and the number of joints with active disease or the ESR. We observed a positive association between NCR− ILC3s and the number of joints with active disease and a weak association between NCR− ILC3s and the ESR (Figures 2F and G; also see Supplementary Table 4). No association was seen between ILC1s and NCR+ ILC3s and either the number of joints with active disease or the ESR. Markedly, no differences were observed in the frequency of ILC1s, NCR− ILC3s, and NCR+ ILC3s within SFMCs between treatment‐naive patients and patients receiving methotrexate (see Supplementary Figures 1B–D, http://onlinelibrary.wiley.com/doi/10.1002/art.40731/abstract).

#### Expansion of NCR− ILC3s in SF of JIA patients is associated with an increase in IL‐17A–producing CD4+ T cells, CD8+ T cells, and CD4−CD8− T cells

We have demonstrated that ILC subset frequency and transcriptional profile are altered in SFMCs isolated from JIA patients, and that among the ILC subsets identified within JIA SFMCs, an expansion of NCR− ILC3s has the strongest association with multiple measures of clinical severity. NCR− ILC3s can be characterized by the expression of the transcripts for *RORC2* and IL‐17A, similar to Th17 cells, and they can be found expanded in IL‐17A–driven pathologies 8. Taken together, these data suggest that the IL‐17A+CD4+ T cell signature we have previously described in JIA SF may extend to other cell types including ILCs 2.

**Figure 3:**
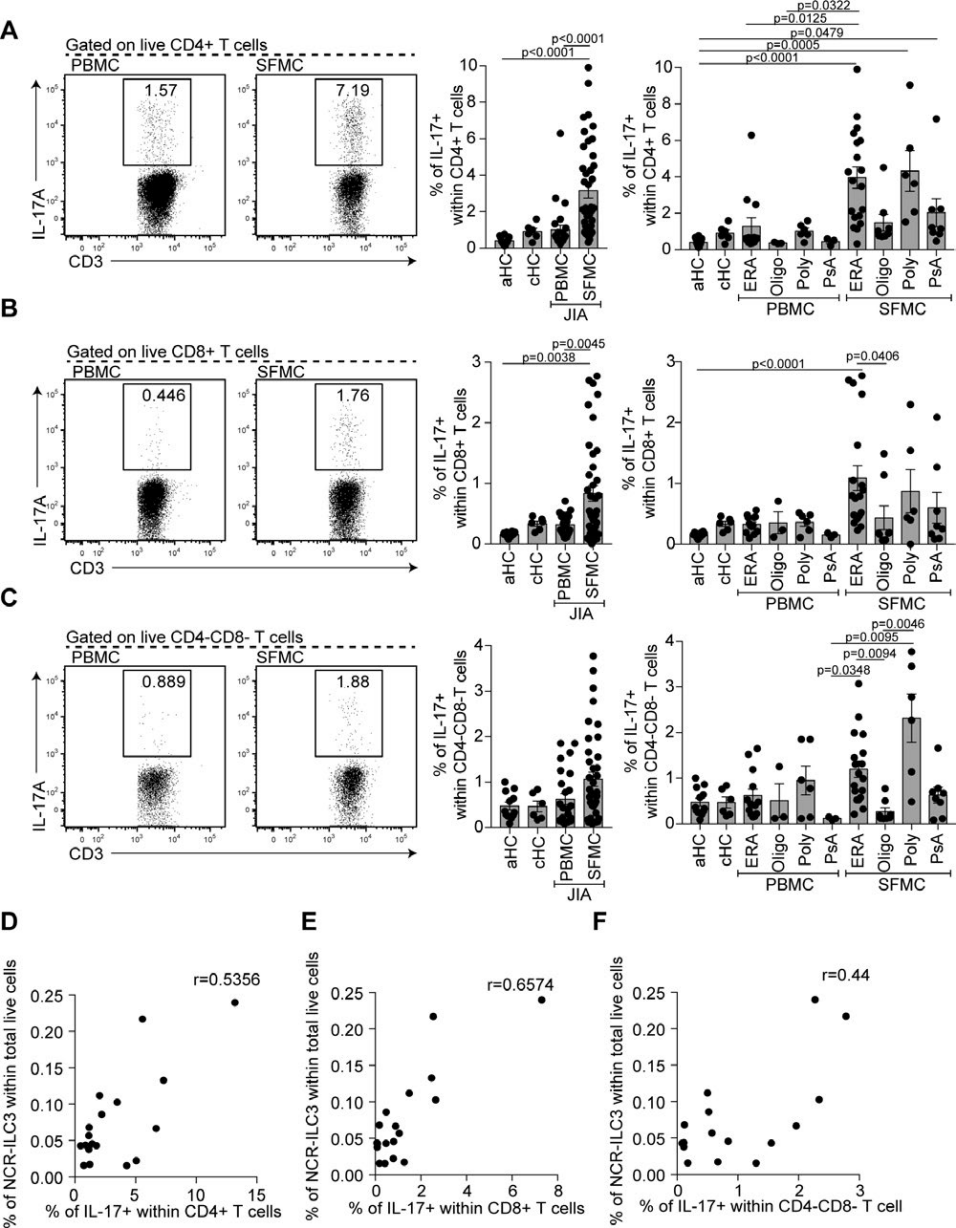
Interleukin‐17A (IL‐17A)–producing CD4+, CD8+, and γδ T cells in SF of patients with JIA. **A**–**C**, Representative flow cytometry plots and summary scatterplots with bar charts showing the frequency of IL‐17A+ cells within CD4+ T cells (**A**), CD8+ T cells (**B**), and CD4−CD8− T cells (**C**) in CD3+ gated live cells in PBMCs from healthy adults (n = 12), PBMCs from healthy children (n = 6), PBMCs from JIA patients (total n = 25; 13 with ERA, 3 with oligoarticular JIA, 6 with polyarticular JIA, and 3 with PsA), and SFMCs from JIA patients (total n = 41; 17 with ERA, 8 with oligoarticular JIA, 6 with polyarticular JIA, and 10 with PsA). Symbols represent individual patients; bars show the mean ± SEM. Significance was determined by Kruskal‐Wallis tests with Dunn's multiple comparison tests. **D**–**F**, Scatterplots showing the relationships of the frequency of IL‐17A+CD4+ T cells (**D**), IL‐17A+CD8+ T cells (**E**), and IL‐17A+CD4−CD8− T cells (**F**) with the percentage of NCR− ILC3s within total live cells within JIA SFMCs (n = 16 patients). Significance was determined by Spearman correlation with Bonferroni correction for multiple testing. See Figure 1 for other definitions.

To investigate this, we quantified other potential IL‐17A–producing cell types; these included CD4+ T cells, CD8+ T cells, and CD4−CD8− T cells. As previously described 2, we found higher percentages of IL‐17A+CD4+ T cells within JIA SFMCs than within healthy control and JIA PBMCs (Figure 3A). Analysis of IL‐17A+CD8+ T cells and IL‐17A+CD4−CD8− T cells (of which ~80% were γδ T cells; data not shown) established that there was also a significantly higher percentage of IL‐17A+CD8+ T cells and a trend toward an increase in IL‐17A+CD4−CD8− T cells in JIA SFMCs compared to healthy control adult PBMCs and JIA PBMCs (Figures 3B and C). No significant differences were seen between healthy control adult PBMCs, healthy control children PBMCs, and JIA PBMCs. Unlike the enrichment of NCR− ILC3s within SFMCs of all subtypes of JIA (Figure 1F), there were significant differences in the frequency of IL‐17A–producing T cells between JIA subtypes. For example, consistent with published reports demonstrating a strong IL‐17A signature in ERA (9), we found an increase in IL‐17A–producing CD4+, CD8+, and CD4−CD8− T cells in SFMCs isolated from patients with ERA (Figures 3A–C). It is worth highlighting, however, that a proportion of patients displayed a strong IL‐17A signature regardless of subtype.

To assess whether the IL‐17A T cell signature was associated with an expansion of NCR− ILC3s, a correlation analysis was performed between the various synovial subpopulations analyzed. Positive correlations were observed in SFMCs between NCR− ILC3s as a proportion of total live cells and IL‐17A+ cells in the CD4+ and CD8+ T cell compartments (Figures 3D and E; also see Supplementary Table 4, http://onlinelibrary.wiley.com/doi/10.1002/art.40731/abstract). A weak potential association with IL‐17A+CD4−CD8− T cells was also observed (Figure 3F; also see Supplementary Table 4). These data demonstrate that the expansion of NCR− ILC3s is concomitant with the expansion of IL‐17A–producing T cells within JIA SFMCs, which indicates that the cell‐specific contribution to the IL‐17A milieu in JIA SFMCs may be a key determinant of clinical outcome. Of note, correlations of the percentages of IL‐17+CD4+ T cells, IL‐17+CD8+ T cells, and IL‐17+CD4−CD8− T cells with the physician's VAS score, number of joints with active disease, and ESR demonstrated that among the T cell subsets, CD4+ T cells had the strongest association with the physician's VAS score (see Supplementary Figure 2 and Supplementary Table 4, http://onlinelibrary.wiley.com/doi/10.1002/art.40731/abstract). However, unlike NCR− ILC3s, the percentages of IL‐17A+ T cell subsets did not have any association with the number of joints with active disease or the ESR.

## Discussion

Recent investigation into ILC biology and function has led to an appreciation of their role in tissue/immune homeostasis and their contribution to immunopathology. However, this is the first study to investigate whether ILC phenotype and function are altered in JIA. We demonstrate that ILC1s, NCR− ILC3s, and NCR+ ILC3s are expanded within JIA SFMCs. Most interestingly, NCR− ILC3s, the innate equivalent to Th17 cells 10, exhibit a positive association with disease severity and with an increase in multiple IL‐17A–producing T cell subsets. Future studies are needed to confirm our hypothesis that the strength of the IL‐17A signature within JIA SFMCs is shared among multiple cell types.

Both human studies and animal models support a central role for IL‐17A in JIA pathogenesis. For example, IL‐17A–deficient mice are resistant to the induction of collagen‐induced arthritis 11, and levels of IL‐17A are known to be significantly higher in JIA SF 9. However, the relative contribution of different cell types to IL‐17A production within the inflamed joint remains relatively unexplored. Our data show that both innate lymphoid cells (NCR− ILC3s) and adaptive lymphoid cells (CD4+, CD8+, and γδ T cells) may contribute to IL‐17A production at the inflammatory site. Further work is needed to unravel the mechanisms that underlie the preferential accumulation of multiple IL‐17A–producing cell types within JIA SFMCs. One possible explanation is that the presence of high levels of IL‐1β, IL‐23, and IL‐6 within the synovial environment in JIA 12 creates an “IL‐17A–skewing” microenvironment that induces the differentiation of Th17 cells and ILC3s 3, 13. Future studies using single‐cell RNA sequencing will aid in defining detailed functional and transcriptional heterogeneity in ILCs within SFMCs, as recently reported for human tonsils 14. At present, our data demonstrate the presence of an inflammatory Th1 cell and Th17 cell transcriptional signature in total ILCs within SFMCs, but not which ILC1/ILC3 subset is responsible for this transcriptional signature. This is especially pertinent as it is not yet known how the inflammatory environment of the arthritic joint alters the plasticity of ILC1/ILC3 subsets.

Despite success of JIA treatment with tumor necrosis factor and IL‐6 blockade, a group of patients remain unresponsive to treatment. Our exploratory analysis demonstrates that disease severity could potentially be associated with an increase in multiple IL‐17–producing lymphoid cell types. This suggests that treatments targeting IL‐17A could be efficacious in a significant proportion of JIA patients (who may fall into several of the current clinically defined subtypes). This notion is further supported by recent evidence that secukinumab, a monoclonal antibody against IL‐17A, is effective in the treatment of ankylosing spondylitis, another IL‐17A–driven autoimmune disease 15. Our observations raise the possibility that patients may be better stratified for treatment with biologic agents based on immune phenotype rather than on previously ascribed clinical categories. It is now essential to gain a whole‐system view of IL‐17A biology in order to design novel therapeutic strategies.

## Author contributions

All authors were involved in drafting the article or revising it critically for important intellectual content, and all authors approved the final version to be published. Dr. Rosser had full access to all of the data in the study and takes responsibility for the integrity of the data and the accuracy of the data analysis.

### Study conception and design

Rosser, Lom, Bending, Bajaj‐Elliott, Wedderburn.

### Acquisition of data

Rosser, Lom, Duurland.

### Analysis and interpretation of data

Rosser, Lom, Bending, Duurland, Bajaj‐Elliott, Wedderburn.
