# Supplementary figures and images for: Innate Lymphoid Cells and T Cells Contribute to the Interleukin‐17A Signature Detected in the Synovial Fluid of Patients With Juvenile Idiopathic Arthritis
Source: Arthritis Rheumatol. 2019 Jan 28;71(3):460–7. doi: 10.1002/art.40731 (PMC7983174; doi:10.1002/art.40731)

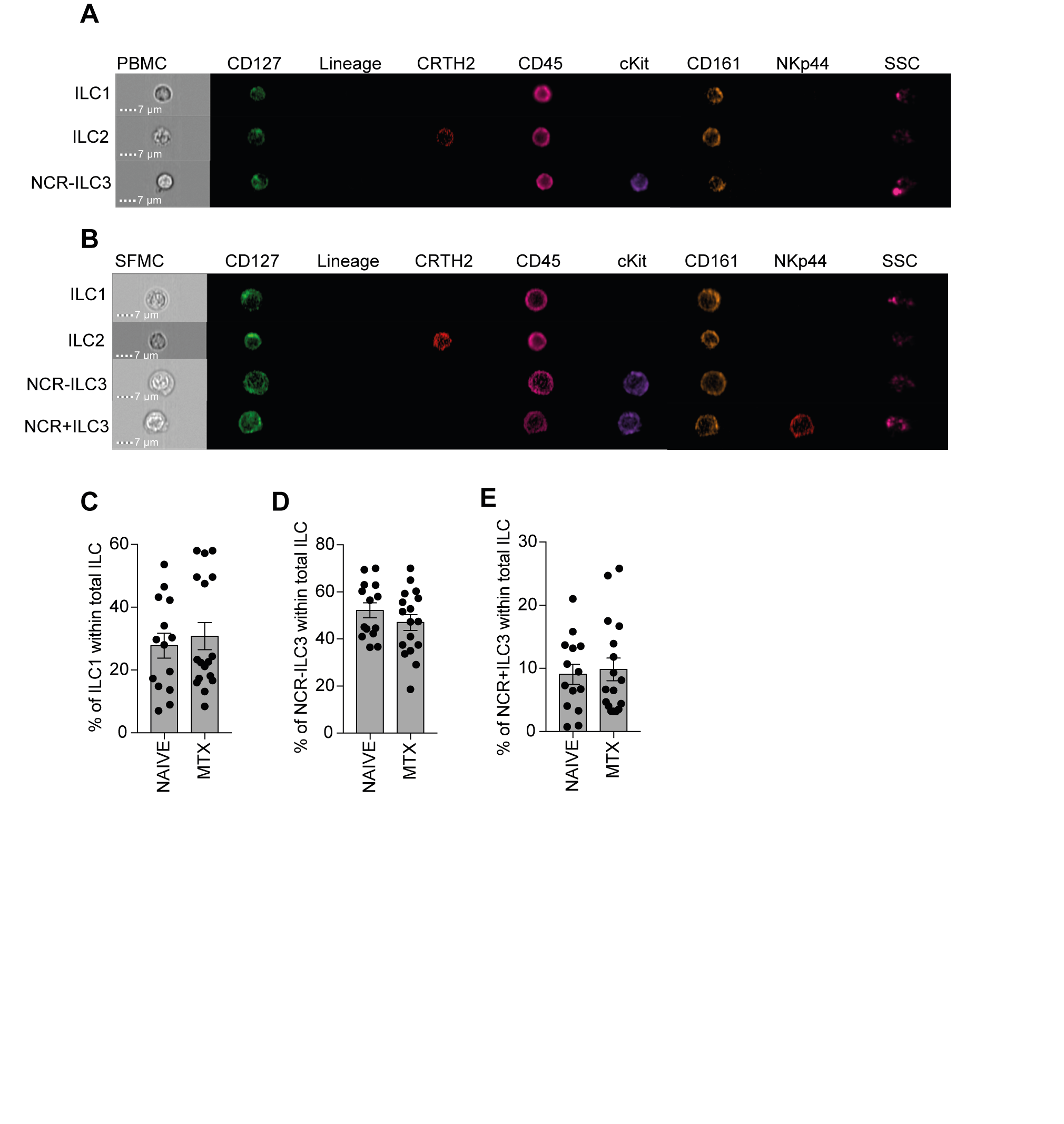

Supplement: Supplementary file 1 [file ART-71-460-s001.tif]

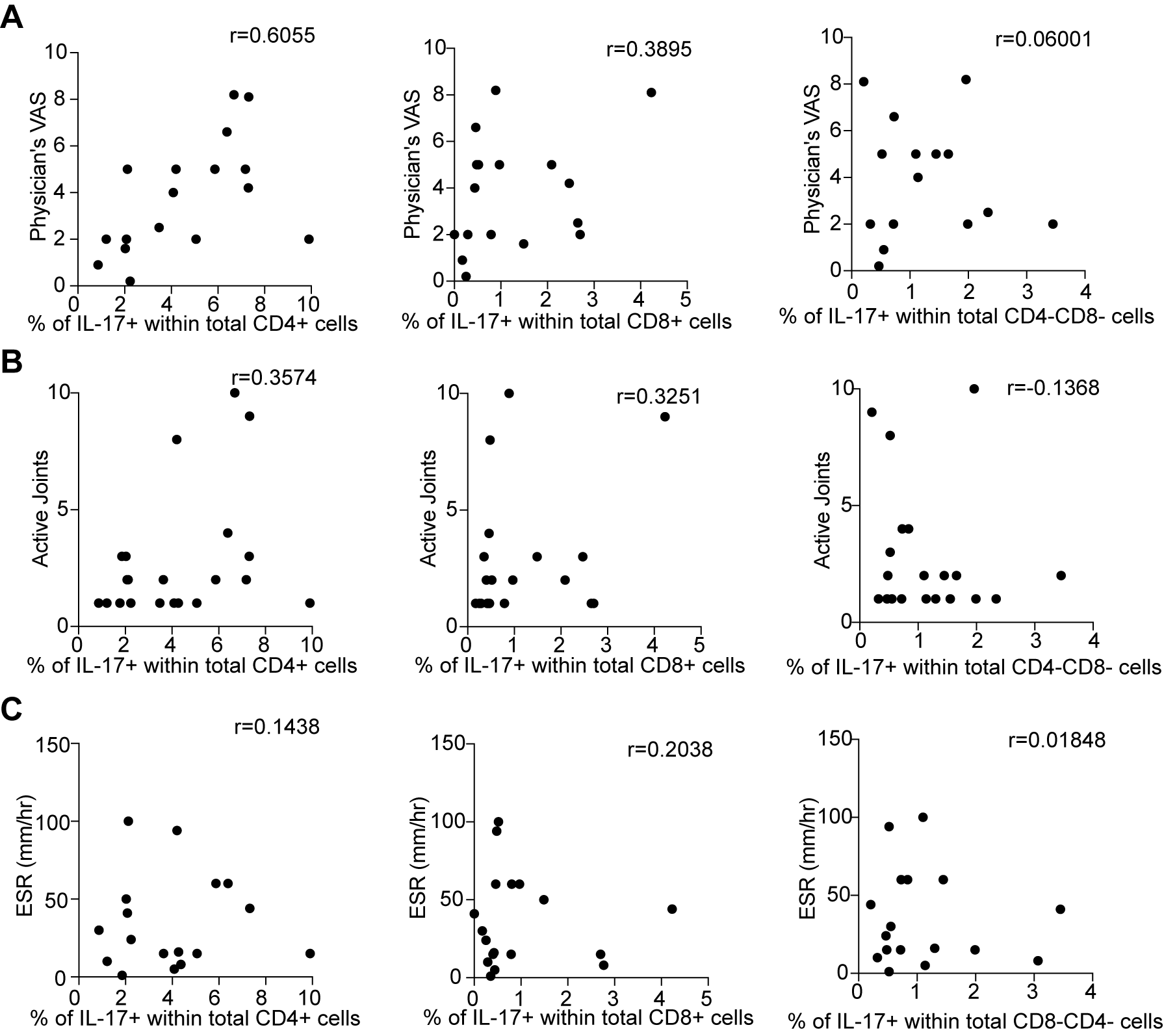

Supplement: Supplementary file 2 [file ART-71-460-s002.tif]
