# Supplementary material for: Innate Lymphoid Cells and T Cells Contribute to the Interleukin‐17A Signature Detected in the Synovial Fluid of Patients With Juvenile Idiopathic Arthritis
Source: Arthritis Rheumatol. 2019 Jan 28;71(3):460–7. doi: 10.1002/art.40731 (PMC7983174; doi:10.1002/art.40731)
Supplement: Supplementary file 3 [file ART-71-460-s003.docx]

**Supplementary Table 1. Patient demographics**

|  | **Adult healthy control**  **(n=22)** | **Child healthy control**  **(n=7)** | **Oligo-arthritis**  **(n=22)** | **Polyarticular arthritis**  **(n=14)** | **Enthesitis related arthritis**  **(n=25)** | **Psoriatic arthritis**  **(n=10)** |
| --- | --- | --- | --- | --- | --- | --- |
| **No. male/female** | 8/14 | 5/2 | 10/12 | 6/8 | 22/3 | 2/8 |
| **Age at sampling (years), median (IQR)** | 25.99  (24.93- 30.19) | 6.29 (5.48 – 8.31) | 8.70  (7 – 11.17) | 10.97  (9.39 – 12.86) | 14.00 (12.00 – 16.46) | 12.32 (11.20 – 13) |
| **Treatment received within preceding 6 months of sample: MTX (%)** | n/a | n/a | 9/22  (41%) | 11/14  (78.57%) | 12/25  (48%) | 4/10  (40%) |
| **Treatment received within preceding 6 months of sample: Biological therapy (%)** | n/a | n/a | 0/22  (0%) | 3/14  (21.42%) | 1/25  (4%) | 2/10  (20%) |
| **No. of swollen/tender joints involved at time of sampling, median (IQR)** | n/a | n/a | 1  (1 -2) | 2  (2 – 3.5) | 2 .50  (1 -4.5) | 2  (1.25 – 4.25) |
| **ESR mm/hr at time of sampling, median (IQR)** | n/a | n/a | 25  (11 – 46) | 41  (12 – 68) | 19  (15 - 60) | 30  (12 – 21) |
| **HLA-B27+ (%)** | n/a | n/a | n/a | n/a | 21/25  (84%) | n/a |
| **RF + (%)** | n/a | n/a | n/a | 2/14  (14.28%) | n/a | n/a |

**Supplementary Table 2. List of Antibodies for Flow Cytometry and Cell Sorting**

| **Marker** | **Fluorochrome** | **Clone** | **Supplier** |
| --- | --- | --- | --- |
| CD1a | PE | H149 | eBiosciences |
| CD3 | PE | UCHT1 | Biolegend |
| CD3 | V500 | UCHT1 | BD Biosciences |
| CD3 | BV605 | OKT3 | BioLegend |
| CD3 | BV711 | OKT3 | BioLegend |
| CD4 | BV711 | OKT4 | BioLegend |
| CD8a | FITC | SK1 | BD Biosciences |
| CD8a | APC | SK1 | eBioscience |
| CD11c | PE | 3.9 | BioLegend |
| CD14 | PE | 61D3 | BioLegend |
| CD16 | PE | B73.1 | eBiosciences |
| CD19 | PE | HIB19 | BioLegend |
| CD34 | PE | 561 | BioLegend |
| CD45 | BV421 | HI30 | BioLegend |
| CD45 | PE-CY7 | HI30 | BioLegend |
| CD94 | PE | DX22 | BioLegend |
| CD123 | PE | 6H6 | BioLegend |
| CD127 | FITC | EBIORDR5 | eBiosciences |
| CD127 | BV711 | A019D5 | BioLegend |
| CD161 | APC | HP-3G10 | eBiosciences |
| CD161 | BV605 | HP-3G10 | BioLegend |
| CD161 | PE-CY7 | HP-3G10 | eBiosciences |
| CD161 | BV421 | HP-3G10 | BioLegend |
| CRTH2 | FITC | BM16 | BioLegend |
| cKit | BV421 | 104D2 | BioLegend |
| BDCA2 | BV510 | 1A4 | BioLegend |
| FcεRIα | PE | AER-37 | BioLegend |
| NKp44 | APC | P44-8 | BioLegend |
| αβTCR | PE | IP26 | BD Biosciences |
| γδTCR | PE | B1 | BD Biosciences |
| IL-17AA | BV605 | BL168 | BioLegend |

**Supplementary Table 3. List of primer sequences for PCR**

| **Gene** | **Primer Sequences 5’- 3’** | | **Annealing Temp (°C)** |
| --- | --- | --- | --- |
| *ACTB* | F | AGA TGAC CCAGATCATGTTTGAG | 60 |
|  | R | AGGTCCA GACGCAG GATG |  |
| *TBX21* | F | CCCCAAG GAATTGAC AGTTG | 60 |
|  | R | GGGAAAC TAAAGCTC ACAAAC |  |
| *IFNG* | F | TGACCAG AGCATCCA AAAGA | 60 |
|  | R | CTCTTCGA CCTCGAAA CAGC |  |
| *GATA3* | F | ACCACAAC CACACTCT GGAGGA | 60 |
|  | R | TCGGTTTC TGGTCTG GATGCCT |  |
| *IL13* | F | A TTGCTCT CACTTGCC TTGG | 60 |
|  | R | GTCAGGTT GATGCTC CATACC |  |
| *RORC* | F | AATCTGGA GCTGGCC TTTCA | 60 |
|  | R | CTGGAAG ATCTGCAG CCTTT |  |
| *AHR* | F | CTTAGGCT CAGCGTC AGTTA | 60 |
|  | R | GTAAGTTC AGGCCTT CTCTG |  |
| *IL17A* | F | AATCTCCA CCGCAAT GAGGA | 60 |
|  | R | ACGTTCCC ATCAGCGT TGA |  |
| *IL22* | F | CCCATCA GCTCCCA CTGC | 60 |
|  | R | GGCACCA CCTCCTG CATATA |  |

**Supplementary Table 4. Uncorrected p values for correlation analyses.**

| **Figure** | **X Axis** | **Y Axis** | **Uncorrected p Value** |
| --- | --- | --- | --- |
| Fig. 2E | % of ILC1 within total live cells | Physician’s VAS | p=0.0179 |
| Fig. 2E | % of NCR-ILC3 within total live cells | Physician’s VAS | p=0.0048 |
| Fig. 2E | % of NCR+ILC3 within total live cells | Physician’s VAS | p=0.0352 |
| Fig. 2F | % of ILC1 within total live cells | Active Joints | p=0.4552 |
| Fig. 2F | % of NCR-ILC3 within total live cells | Active Joints | p=0.0055 |
| Fig. 2F | % of NCR+ILC3 within total live cells | Active Joints | p=0.8539 |
| Fig. 2G | % of ILC1 within total live cells | ESR (mm/hr) | p=0.2307 |
| Fig. 2G | % of NCR-ILC3 within total live cells | ESR (mm/hr) | p=0.0830 |
| Fig. 2G | % of NCR+ILC3 within total live cells | ESR (mm/hr) | p=0.1599 |
| Fig. 3D | % of IL-17+ within CD4+ T cells | % of NCR-ILC3 within total live cells | p=0.0220 |
| Fig. 3E | % of IL-17+ within CD8+ T cells | % of NCR-ILC3 within total live cells | p=0.0030 |
| Fig. 3F | % of IL-17+ within CD4-CD8- T cells | % of NCR-ILC3 within total live cells | p=0.0893 |
| Sup Fig. 2A | % of IL-17+ within CD4+ T cells | Physician’s VAS | p=0.0114 |
| Sup Fig. 2A | % of IL-17+ within CD8+ T cells | Physician’s VAS | p=0.1263 |
| Sup Fig. 2A | % of IL-17+ within CD4-CD8- T cells | Physician’s VAS | p=0.8320 |
| Sup Fig. 2B | % of IL-17+ within CD4+ T cells | Active Joints | p=0.1117 |
| Sup Fig. 2B | % of IL-17+ within CD8+ T cells | Active Joints | p=0.1620 |
| Sup Fig. 2B | % of IL-17+ within CD4-CD8- T cells | Active Joints | p=0.5651 |
| Sup Fig. 2C | % of IL-17+ within CD4+ T cells | ESR (mm/hr) | p=0.5818 |
| Sup Fig. 2C | % of IL-17+ within CD8+ T cells | ESR (mm/hr) | p=0.4069 |
| Sup Fig. 2C | % of IL-17+ within CD4-CD8- T cells | ESR (mm/hr) | p=0.9448 |

P values below 0.0023 are considered statistically significant once corrected with Bonferroni’s post-test for multiple testing.
