# Supplementary material for: Innate Lymphoid Cells and T Cells Contribute to the Interleukin‐17A Signature Detected in the Synovial Fluid of Patients With Juvenile Idiopathic Arthritis
Source: Arthritis Rheumatol. 2019 Jan 28;71(3):460–7. doi: 10.1002/art.40731 (PMC7983174; doi:10.1002/art.40731)
Supplement: Supplementary file 4 [file ART-71-460-s004.doc]

**Supplementary Figure 1. SFMC-ILC subsets have the same morphology as PBMC-ILC subsets and their frequency are not altered by treatment status.** ILC subsets were identified according to fluorescence of subset specific protein expression by analysis of individual cell images as shown using image stream. Representative images of ILC subsets from (A) PBMC and (B) SFMC from JIA patients. All ILC detected had a characteristically dense single nuclei, a thin halo of perinuclear cytoplasm, were approx 7μm and had cell markers restricted to the cell surface. Left hand panel (grey) shows the brightfield image. Summary scatter plots with bar charts showing the frequency of (C) ILC1, (B) NCR-ILC3 and (E) NCR+ILC3 with JIA-SFMC of treatment naïve JIA patients 9n=14) and patients on methotrexate (n=17). Bar charts represent mean ± SE.

**Supplementary Figure 2. IL-17A+ T cell subsets can be associated with clinical measure of disease severity.** Scatter plots showing relationship between (A) physician’s VAS (n=15), (B) active joints (n=21) and (C) ESR (n=17) and the frequency of IL-17+CD4+ T cells, IL-17+CD8+ T cells and IL-17+CD4-CD8- T cells within JIA-SFMC. Statistical analysis carried out by Spearman correlation analysis with Bonferroni’s correction for multiple testing.
